# A 3-year prospective study of the epidemiology of acute respiratory viral infections in hospitalized children in Shenzhen, China

**DOI:** 10.1111/irv.12257

**Published:** 2014-05-14

**Authors:** Ying He, Guang-Yu Lin, Qiong Wang, Xiao-Ying Cai, Yin-Hui Zhang, Chuang-Xing Lin, Chang-Dong Lu, Xue-Dong Lu

**Affiliations:** a Department of Laboratory Medicine, Affiliated Futian People's Hospital, Guangdong Medical CollegeShenzhen, China; b Institute of Clinical Microbiology, Affiliated Futian People's Hospital, Guangdong Medical CollegeShenzhen, China; c Department of Paediatrics, Affiliated Second Hospital, Medical College of Shantou UniversityShantou, China; d Department of Paediatrics, Affiliated Futian People's Hospital, Guangdong Medical CollegeShenzhen, China

**Keywords:** Acute respiratory tract infection, China, hospitalized children, viruses

## Abstract

**Background:**

The epidemiology of local viral etiologies is essential for the management of viral respiratory tract infections. Limited data are available in China to describe the epidemiology of viral respiratory infections, especially in small–medium cities and rural areas.

**Objectives:**

To determine the viral etiology and seasonality of acute respiratory infections in hospitalized children, a 3-year study was conducted in Shenzhen, China.

**Methods:**

Nasopharyngeal aspirates from eligible children were collected. Influenza and other respiratory viruses were tested by molecular assays simultaneously. Data were analyzed to describe the frequency and seasonality.

**Results:**

Of the 2025 children enrolled in the study, 971 (48·0%) were positive for at least one viral pathogen, in which 890 (91·7%) were <4 years of age. The three most prevalent viruses were influenza A (IAV; 35·8%), respiratory syncytial virus (RSV; 30·5%) and human rhinovirus (HRV; 21·5%). Co-infections were found in 302 cases (31·1%), and dual viral infection was dominant. RSV, HRV and IAV were the most frequent viral agents involved in co-infection. On the whole, the obvious seasonal peaks mainly from March to May were observed with peak strength varying from 1 year to another.

**Conclusions:**

This study provides a basic profile of the epidemiology of acute respiratory viral infection in hospitalized children in Shenzhen. The spectrum of viruses in the study site is similar to that in other places, but the seasonality is closely related to geographic position, different from that in big cities in northern China and neighboring Hong Kong.

## Introduction

Acute respiratory tract infections (ARTIs) are a persistent and pervasive public health problem in both developed and developing countries. They cause a great burden of disease worldwide. Especially in developing countries including China, ARTIs, mainly pneumonia, are the leading cause of death among children under the age of 5 years.^1^,^2^ A great variety of pathogens can cause ARTIs, and viruses have been considered as the predominant pathogens in this children population.^3^,^4^ The most frequently reported viruses include respiratory syncytial virus (RSV), influenza viruses A and B (IAV, IBV), parainfluenza viruses (PIVs), human rhinovirus (HRV) and adenovirus (ADV), which are responsible for most episodes of ARTIs in children.^1^ In the past decade, several new viruses associated with ARTIs such as human metapneumovirus (HMPV), novel strains of coronaviruses (SARS-CoV, HCoV-NL63 and HKUI), human bocavirus (BOV), WU polyomavirus (WUPoyV) and KI polyomavirus (KIPoyV) have been discovered in human respiratory tract specimens. Among them, some have been identified to be causative pathogens of ARTIs.^1^,^4^,^5^

Currently, there are no approved vaccines or medications available for most of the respiratory viruses.^1^ A better understanding of the epidemiology of viral respiratory tract infections in children plays a key role for the prevention, control and treatment of ARTIs. Studies showed that many viral respiratory infections exhibited predictable seasonal variations. However, the epidemiological profiles of viral respiratory infections from different climate zones or different countries in the same climate zone may be varied.^6^–^12^

China is a large country crossing three climate zones, and great differences in climate are found from region to region. A better understanding of the epidemiology of ARTIs in different regions could be helpful to develop effective surveillance, prevention and treatment strategies. Although some studies on the epidemiology of ARTIs have recently been reported in big cities such as Beijing, Shanghai and Hong Kong,^13^–^16^ the epidemic characteristics of viruses in ARTIs are still not well established all around China, especially in other cities and rural areas.

Shenzhen is the largest migratory city of China with high population density and population mobility. It is located in southern China at 22°27′–22°52′N and 113°46′–114°37′E, immediately north of Hong Kong, with a typical subtropical monsoon climate. The annual average temperature and relative humidity of Shenzhen are about 23°C (12–33°C) and 77%, respectively. The purpose of this study is to investigate the prevalence, seasonality and clinical characteristics of acute viral respiratory infections in hospitalized children in Shenzhen and to provide insights into etiologies of ARTIs in local infants and children.

## Materials and methods

### Study design

A consecutive 3-year prospective study from July 2007 to June 2010 was conducted in Shenzhen, a coastal city neighboring Hong Kong. Four hospitals including a children's hospital were chosen for the study. Selected patients with ARTIs admitted to the pediatric wards were enrolled. The inclusion criteria were as follows: less than 14 years old, acute fever (T ≥ 38°C), with any one of respiratory symptoms (such as sore throat, cough, wheezing and dyspnoea/tachypnoea), normal or low leukocyte count, the onset of illness within 3 days before hospitalization. The diagnosis of pneumonia was based on the guideline of the management of childhood community acquired pneumonia (CAP) issued by the Chinese Medical Association in 2006.^17^ In the guideline, the clinical symptoms and signs for the diagnosis of childhood CAP include fever, cough, tachypnoea (defined according to different age), difficulty breathing and/or lower chest wall indrawing. X-ray evaluation has been carried out when necessary. The study protocol was approved by the medical ethical committees of the hospitals. Written informed consent was obtained from the parents or legal guardians of the children.

### Specimen collection and processing

Nasopharyngeal aspirates (NPA) were obtained by trained personnel following standard operating procedures within 24 hour after admission. The specimens were transported immediately to the laboratory by sterile viral transport media, then divided into aliquots and immediately frozen at −80°C until further processing.

Total viral nucleic acids (DNA and RNA) were extracted from 200 μl of NPA specimen using the AxyPrep Body Fluid DNA/RNA Miniprep Kit (Axygen, Union City, CA, USA), according to the manufacturer's instructions. Purified DNA and RNA were stored at −80°C in aliquots for further PCR analysis.

### Molecular detection of viruses

For each specimen, assays for ten common and newly identified viruses were performed. Briefly, WUPoyV and BOV were tested using monoplex PCRs described previously.^18^,^19^ Other viruses were tested using the Luminex platform and multiplex xTAG™ respiratory viral panel assay (RVP Assay) according to the manufacturer's instructions.^20^ All multiple infection samples were retested. If there was discordance between two tests, the sample was confirmed by monoplex PCR.

### Statistical analysis

Statistical Package for the Social Sciences (SPSS) for Windows version 11.0 (SPSS Inc. Chicago, IL, USA) was used. For comparison of categorical data, chi-square or Fisher's exact test was used. All tests were two-tailed, and a *P* value below 0·05 was considered statistically significant.

## Results

### Patient characteristics and clinical diagnosis

A total of 2025 specimens were obtained from 2025 eligible patients ranging from 15 days to 14 years old with a median age of 12 months, in which 89·6% of patients were < 4 years old. There were 964 (47·6%) females and 1061 (52·4%) males included. Of all hospitalized children enrolled in this study, 84·0% involved lower respiratory infection and 16·0% had upper respiratory tract infection (Table1). Among 971 positive cases, 572 (58·9%) were diagnosed as pneumonia.

**Table 1:** Clinical diagnosis and respiratory viruses detected in all 2025 ARTI cases

	Single infection										
	
	RSV	IAV	HRV	BOV	PIV3	ADV	HMPV	WUPoyV	PIV1	IBV	Total
A											
Total cases	163	172	85	55	51	49	37	26	24	7	669
Bronchitis	12	19	7	7	5	2	2	3	5	0	62
Bronchiolitis	45	19	20	7	13	9	8	2	5	0	128
Pneumonia	93	97	55	28	24	28	25	18	14	5	387
URTI	13	37	3	13	9	10	2	3	0	2	92

	Positive			Negative	Total
	
	Single infection	Co-infection	Subtotal		
B					
Total cases	669	302	971	1054	2025
Bronchitis	62	21	83	112	195
Bronchiolitis	128	54	182	173	355
Pneumonia	387	185	572	579	1151
URTI	92	42	134	190	324

URTI, upper respiratory tract infection; RSV, respiratory syncytial virus; IVA, influenza virus A; IBV, influenza virus B; HRV, human rhinovirus; BOV, human bocavirus; ADV, adenovirus; HMPV, human metapneumovirus; WUPoyV, WU polyomavirus.

### Overall detection of viruses

About 971 of the 2025 cases (48·0%) were positive for at least one viral pathogen. Among them, IAV, RSV, HRV and PIVs were detected in 348 (35·8%), 296 (30·5%), 209 (21·5%) and 169 (17·4%) cases, respectively. Single infection was observed in 669 (68·9%) cases, and multiple infection was found in 302 (31·1%). Our results also showed that RSV, IAV and HRV were the main pathogens in single viral infection cases (Table1).

The overall positive rates in the year of 2007–2008, 2008–2009 and 2009–2010 were 43·3%, 52·5% and 46·4%, respectively. It was statistically higher in the year of 2008–2009 than in the other 2 years (χ^2^ = 10·18, *P* = 0·0014), but no difference was revealed between the years of 2007–2008 and 2009–2010 (χ^2^ = 1·191, *P* = 0·2752).

**Figure 1:**
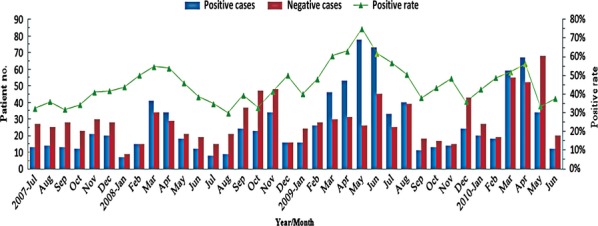
Monthly distribution and positive rates of acute respiratory tract infection cases in 2025 inpatient children, July 2007–June 2010.

The monthly positive rates varied from 32·5% to 75·0% with a mean of 45·1% (Figure1). In the year 2009, when influenza A (H1N1) was pandemic worldwide, the positive rate started to increase in March and the highest positive rate 75·0% was observed in May.

Among the 971 positive cases, a total of 1335 viral pathogens were detected. The most frequently detected pathogen was IAV (26·1%, 348/1335), followed by RSV (22·2%, 296/1335), HRV (15·7%, 209/1335), PIV1 and PIV3 (12·7%, 169/1335) (Table2). Among all 348 cases with IAV infection, 200 (57·7%) were hospitalized from March to August 2009.

**Table 2:** The distribution and infection patterns of viral agents according to age groups

	≤6 months, *n* = 595		6 months –1 years, *n* = 408		1–2 years, *n* = 408		2–4 years, *n* = 404		4–6 years, *n* = 114		>6 years, *n* = 96		All ages, *n* = 2025	
							
	No. (%)	Mixed	No. (%)	Mixed	No. (%)	Mixed	No. (%)	Mixed	No. (%)	Mixed	No. (%)	Mixed	No. (%)	Mixed
Positive	300 (14·8)*^,^**		182 (9·0)***		211 (10·4)***		197 (9·7)***		48 (2·4)†		33 (1·6)†		971 (48·0)	
Single	213 (21·9)††		126 (13·0)		146 (15·0)		129 (13·3)		32 (3·3)		23 (2·4)		669 (68·9)	
Mixed	87 (9·0)††		56 (5·8)		65 (6·7)		68 (7·0)		16 (1·6)		10 (1·0)		302 (31·1)	
Negative	295 (14·6)*		226 (11·2)		197 (9·7)		207 (10·2)		66 (3·3)		63 (3·1)		1054 (52·0)	
RSV	109 (11·2)†††	45 (41·3)‡	54 (5·6)	27 (50·0)	57 (5·9)	24 (42·1)	60 (6·2)	27 (45·0)	8 (0·8)	5 (62·5)	8 (0·8)	5 (62·5)	296 (30·5)	133 (44·9)
IAV	97 (10·0)	58 (59·8)	55 (5·7)	32 (58·2)	90 (9·3)	38 (42·2)	68 (7·0)	36 (52·9)	25 (2·6)	9 (36·0)	13 (1·3)	3 (23·1)	348 (35·8)	176 (50·6)
IBV	6 (0·6)	4 (66·7)	3 (0·3)	2 (66·7)	3 (0·3)	1 (33·3)	7 (0·7)	5 (71·4)	2 (0·2)	2 (100·0)	0 (0·0)	0 (0·0)	21 (2·2)	14 (66·7)
HRV	66 (6·8)	34 (51·5)	45 (4·6)	25 (55·6)	44 (4·5)	27 (61·4)	38 (3·9)	28 (73·7)	9 (0·9)	5 (55·6)	7 (0·7)	5 (71·4)	209 (21·5)	124 (59·3)
PIV1	15 (1·5)	8 (53·3)	14 (1·4)	9 (64·3)	11 (1·1)	5 (45·5)	13 (1·3)	8 (61·5)	4 (0·4)	3 (75·0)	2 (0·2)	2 (100·0)	59 (6·1)	35 (59·3)
PIV3	32 (3·3)	13 (40·6)	23 (2·4)	9 (39·1)	26 (2·7)	17 (65·4)	24 (2·5)	18 (75·0)	5 (0·5)	2 (40·0)	0 (0·0)	0 (0·0)	110 (11·3)	59 (53·6)
BOV	23 (2·4)	13 (56·5)	22 (2·3)	6 (27·3)	26 (2·7)	13 (50·0)	19 (2·0)	7 (36·8)	6 (0·6)	4 (66·7)	4 (0·4)	2 (50·0)	100 (10·3)	45 (45·0)
ADV	25 (2·6)	7 (28·0)	20 (2·1)	9 (45·0)	14 (1·4)	9 (64·3)	16 (1·6)	6 (37·5)	5 (0·5)	4 (80·0)	9 (0·9)	5 (55·6)	89 (9·2)	40 (44·9)
HMPV	21 (2·2)	10 (47·6)	12 (1·2)	5 (41·7)	9 (0·9)	2 (22·2)	17 (1·8)	7 (41·2)	4 (0·4)	3 (75·0)	1 (0·1)	0 (0·0)	64 (6·6)	27 (42·2)
WUPoyV	11 (1·1)	2 (18·2)	5 (0·5)	2 (40·0)	6 (0·6)	3 (50·0)	14 (1·4)	5 (35·7)	2 (0·2)	1 (50·0)	1 (0·1)	0 (0·0)	39 (4·0)	13 (33·3)
Episodes	405 (41·7)	194 (47·9)	253 (26·1)	126 (49·8)	286 (29·5)	139 (48·6)	276 (28·4)	148 (53·6)	70 (7·2)	38 (54·3)	45 (4·6)	22 (48·9)	1335 (-)	666 (49·9)

RSV, respiratory syncytial virus; IVA, influenza virus A; IBV, influenza virus B; HRV, human rhinovirus; BOV, human bocavirus; ADV, adenovirus; HMPV, human metapneumovirus; WUPoyV, WU polyomavirus.

* Case number and percentage in all enrolled children.

** Incidence rate in this age group significantly higher than the other age groups.

*** No significant difference between these three age groups.

† No significant differences between these two age groups, but significantly lower than the other age groups.

†† Case number and percentage in all positive patients.

††† Episodes of virus detected and percentage in all positive cases.

‡ Co-infection cases of each virus detected and percentages in all cases involving virus.

### Co-infection of viruses

**Figure 2:**
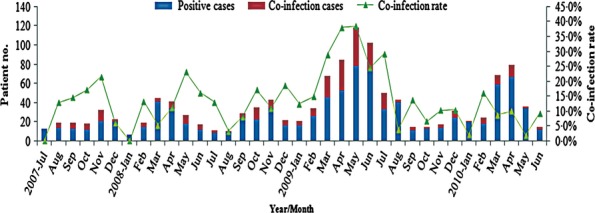
Monthly distribution of co-infection cases and co-infection rates in 2025 inpatient children, July 2007–June 2010.

About 302 co-infection cases were identified, accounting for 14·9% of all 2025 hospitalized children. During the H1N1 outbreak from March to August 2009, co-infection cases and co-infection rate increased significantly (Figure2). 143 of 302 (47·4%) co-infection cases were detected during that time. Among them, 121 cases were involved in IAV infection, including 90 dual infection cases. Of all co-infection cases, 247 (81·8%), 49 (16·2%) and 5 (1·7%) were infected with two, three and four potential viral pathogens, respectively. One multiple infection with five viruses was detected in a 6-month-old infant. IAV, RSV and HRV were the three most frequently found viruses in co-infection and detected in 176, 133 and 124 cases with co-infection rates of 50·6%, 44·9% and 59·3%, respectively (Table2). Various multiple infection patterns were observed in the study. A total of 152 (50·3%) co-infection cases involved at least two viruses of RSV, HRV and IAV. Co-infection rate of each individual virus detected varied significantly. The lowest and highest co-infection rates were observed in WUPoyV (33·3%) and IBV (66·7%), respectively.

91·4% (276/302) of co-infection cases were tested in the age group of 4 years old or younger (Table2), but among all age groups, no statistical difference in co-infection rate was found (χ^2^ = 1·83, *P* = 0·8721). Gender-specific difference in co-infection rate was not observed (χ^2^ = 2·17, *P* = 0·1404). There was no significant difference in co-infection rates between PICU and non-PICU cases. Similarly, no significant difference in clinical symptoms was observed between co-infection and single cases (data not shown).

### Seasonality of viral infections

In general, respiratory viruses were detected more often in the period of March to May than in other months (55·4% and 40·6%, respectively, χ^2^ = 28·06, *P* = 0·0000), and obvious seasonal peaks were observed during those months with peak strength varying from 1 year to another. A weaker seasonal peak could also be distinguished in some winter months in different years (Figure1).

**Figure 3:**
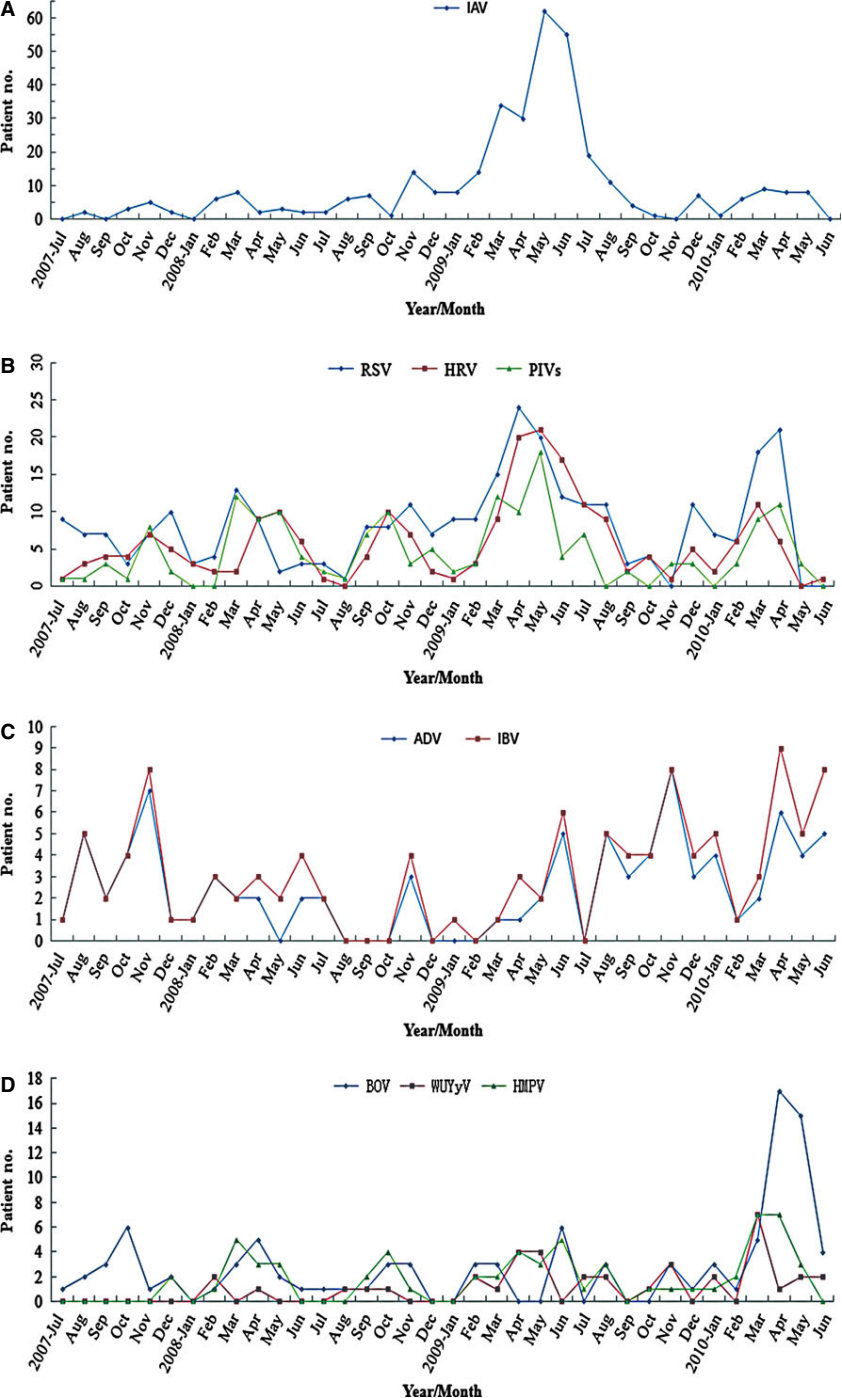
Monthly distribution of single virus detected in 2025 inpatient children, July 2007–June 2010. (A) Influenza virus A (IAV); (B) respiratory syncytial virus (RSV), human rhinovirus (HRV) and parainfluenza viruses (PIVs); (C) Influenza virus B (IBV) and adenovirus (ADV); (D) human bocavirus (HBoV), human metapneumovirus (HMPV), WU polyomavirus (WUPoyV).

The seasonality profile of each individual virus detected was diversified. A seasonal distribution of IAV can be observed from late spring to summer (mainly March to May) and sometimes in fall (October, November or December). A wide seasonal peak of IAV infection was detected from March to August 2009 (Figure3A). Although RSV was tested almost a whole year, two yearly peaks were identified. One was found in November and/or December and the other stronger one was found in March to May of the year. The peak duration in 2009 was longer than those in other years. The seasonal trends of HRV and PIVs were similar to that of RSV, but the peaks of these three viruses fluctuated and shifted mildly (Figure3B). Although IBV and ADV had a low detection rate in the study, similar seasonality was observed and their infection peaks were mainly in mid-winter. Peaks in spring and summer were also observed in some years (Figure3C).

Our investigation did not find regular seasonality in BOV infections. A sudden increase in BOV infection was recorded in April and May 2010. Although the positive rate of HMPV infection was only 4·8%, regular seasonality was observed from March to May of each year. Of 39 patients with WUPoyV infection, 36 were detected after July 2008. Our data implied that peak months of WUPoyV infection were from March to May (Figure3D).

### Impact of age and gender on virus detection

The positive rates of viral infections in male and female were 52·5% and 47·5%, respectively. No significant gender difference was revealed (χ^2^ = 0·012, *P* = 0·9118). The distribution of viral agents and infection patterns in different age groups are shown in Table2.

Of all 971 positive children, 890 (91·7%) were 4 years old or younger. The positive rate in this age group was significantly higher than that in children more than 4 years old (χ^2^ = 8·26, *P* = 0·0041). Children under 6 months were the most susceptible to respiratory viral pathogens with a positive rate of 14·8% (Table2).

## Discussion

Very few long-term prospective studies were performed for viral etiologies of ARTIs among hospitalized children. In this present study, the infection frequency, seasonality, co-infection pattern and clinical features of viral respiratory infections were investigated based on prospective analysis of three consecutive year's data from hospitalized children with ARTIs. Our results provided a distinctive epidemiological profile of viral respiratory infections in hospitalized children with ARTIs in the study areas, which was different from those in the big cities in northern China such as Beijing and Shanghai and also different from that in adjacent Hong Kong.

Overall, 48·0% of our cases were positive for respiratory virus infections, which resembled the latest study in the same city.^21^ A similar incidence rate has been obtained in neighboring regions^13^,^22^ and other cities such as Rome^23^ and Milan,^24^ but it was different from other studies.^10^–^12^ In China, the overall positive rate reported varied from 27·3 to 74·8% depending on different areas and detection methods.^15^,^16^,^25^–^31^ The rate of respiratory viral infections varied worldwide, and many factors such as geographic distribution, study design and detection protocols could lead to these variations.^1^,^7^,^8^,^32^ In our study, leukocyte count was used as an indicator of inclusion criteria and it probably affected the positive rate. Viruses not considered in the study, for example coronaviruses, would underestimate the positive rate.

Most studies showed that RSV or HRV was the most prevalent viruses in children with viral respiratory tract infection.^1^ In this study, IAV was the most frequently detected respiratory virus, followed by RSV and HRV. IAV (H1N1) outbreak in 2009 could explain this shift. Data showed that about 60% of IAV infections were detected during the outbreak period. Studies showed that the H1N1 outbreak could change viral distribution patterns.^24^,^29^,^33^ Regardless of the IAV (H1N1) outbreak, RSV and HRV were the two most common viral pathogens in ARTIs, which was consistent with most previous studies.^1^,^10^,^15^,^16^,^22^,^25^–^29^ Our study further confirmed the importance of RSV and HRV in children with ARTIs, especially in children < 4 years of age.^10^,^14^,^23^

Our results also showed that 12·7% of viral pathogens detected were PIV1 and PIV3, which implied that PIVs played an important role in children with ARTIs. Similar findings were obtained in the studies conducted in Shanghai,^14^,^34^ Changsha,^26^ Harbin,^30^ Hong Kong^13^ and Rome.^23^ The prevalence of PIV3 was twofold higher than that of PIV1, particularly in infants, which was similar with other reports,^25^,^26^,^30^,^35^ implying that infants could be more vulnerable to infection with PIV3 than PIV1.

HMPV has been proven to be one of the main viral pathogens responsible for ARTIs in children.^5^ The positive rate found in the study was consistent with previously published results.^10^,^36^,^37^ In China, the infection rate of HMPV varied from 3·2 to 10·6%.^22^,^26^,^28^,^29^,^31^ The seasonality of HMPV in this study was mainly from March to May, similar to that in Hong Kong,^36^ but different from other places.^5^,^37^ In our study, 4·9% of cases were positive for BOV, which coincided with 5·0% in Hong Kong^38^ and higher than Guangzhou^39^ and eastern Guangdong.^22^ Our result suggested that BOV might be present throughout the year with no seasonal distribution. However, seasonal distribution was noted from September to February in Hong Kong^38^ and May and June in Guangzhou.^39^

The use of multiple PCR made it possible to simultaneously detect a broad spectrum of viruses with excellent sensitivity, at the same time, with increased viral detection rate and co-infection rate for ARTIs.^12^,^40^ Among our positive cases, co-infection rate was 31·1%, which was similar to 27·9% reported by Do *et al*.^10^ Co-infection rate reported elsewhere varied widely from 25·4 to 47·9%.^40^ The relatively lower co-infection rates ranging from 0·24 to 26·9% were reported in the studies conducted in various cities of China.^22^,^25^–^31^ In most of these studies, immunofluorescence kits were used to test a lower number of respiratory viruses. It was worth to note that in the study by Peng *et al*.^32^ in Wuhan, China, 69·5% of co-infection rate was reported with immunofluorescence kit. These variations might be attributed to geographic differences, diagnostic methods for viral agents and study design.^12^,^32^,^34^,^41^,^42^ Pathogens in those negative patients need to be further investigated as only ten common and newly identified viruses were included in our study, which might underestimate positive rate or co-infection rate. It was notable that the correlation between co-infection rate and positive rate was not observed.

Of multiple infections, dual infection was predominant in this study whether or not considering the IAV (H1N1) outbreak in 2009, which was consistent with previous studies.^28^,^32^,^42^,^43^ Similar with the studies conducted in the cities of Guangzhou and Wuhan, China,^28^,^29^ our study showed that IAV, RSV and HRV were the main viruses involved in multiple infections. High co-infection rate between these three viruses could be explained from the overlap of their seasonal distributions. A variety of predominant multiple infection patterns between respiratory viruses were observed in different studies.^12^,^32^,^42^,^43^ For example, it was shown in Martin *et al*.'s study^43^ that ADV and coronaviruses were the most common co-infection pattern. Our study showed that RSV and HRV were the two most viruses involved in multiple infection, followed by IAV and PIVs, regardless of IAV infection in the H1N1 outbreak period. It was difficult to explain the variations of co-infection patterns based only on seasonal distribution. A recent study suggested that co-infection patterns were not random and certain pathogens had higher frequency of co-infection.^41^ As molecular assays only detect nucleic acid and positive result does not mean the presence of the pathogen, when studying co-infection patterns of respiratory viruses, the ability to differentiate the real causative pathogens needs to be solved first. Viral load detection could provide some clues for solving this issue.^43^,^44^

Although high co-infection rates have been reported in various studies, the associations among multiple infections, hospitalization rate and severity of ARTIs were still not clear with inconsistent results in different studies.^42^,^43^,^45^ Our data suggested that multiple infection had less association with the severity of disease, consistent with Peng *et al*.'s study.^32^ The relationship between co-infection rates and age group was also investigated in our study, and little correlation was observed. Several previous studies observed that co-infection rates were more frequent in a certain age group, but results were varied.^32^,^43^

In contrast to temperate region, where most viruses had winter-spring seasonality, the respiratory viral infections in tropical and subtropical regions appeared mainly to be spring-summer seasonality.^9^ In this study, due to the high detection rate and similar seasonality of RSV, HRV, IAV, PIV and HMPV, an overall spring-summer seasonality of viral respiratory infections in children was concluded. Studies conducted in Hong Kong showed that a clear seasonal peak was from April to September,^36^,^46^ with a longer duration than our study. The overall seasonality in this study was also different from the studies conducted in northern or central cities of China, in which the seasonality of most viruses presented in autumn-winter and/or winter-spring.^15^,^25^–^27^,^30^ The winter-spring seasonality was also observed in Guangzhou, a city about 150 kilometers north of Shenzhen.^28^ Different seasonal onset and duration were observed in various studies conducted in (sub-) tropical regions. In these studies, ambient temperature, humidity and rainfall were widely used to explain these differences in seasonality, but inconsistent results were observed.^9^,^46^,^47^ Although most studies demonstrated that the seasonality of viral respiratory infections was correlated with increased rainfall, effects of climate factors such as humidity and temperature on the seasonality were complex and interactive.^9^,^46^,^48^ The study areas have four indistinct seasons, and the coldest month usually emerges in January (average 12°C). During the period from March to May, the weather featured warm ambient temperature (average 18–25°C), high relative humidity (average 85%-90%) and increasing rainfall. These meteorological conditions were perhaps conducive to viral survival.^9^,^48^ In addition, intensive temperature fluctuations during seasonal alternation could increase the susceptibility to infections.^49^

As reported in other studies in temperate, tropical and subtropical regions, viral infection rates in children population showed an inverse correlation with age, with younger individuals experiencing higher viral infection rates.^3^,^4^,^6^,^9^,^24^ Our results suggested that children younger than 4 years of age, particularly <6 months, were at higher risk of hospitalization for ARTIs, compared with older children. This was particularly substantiated in RSV infection. Our presumption was supported by other studies.^14^,^25^–^28^ Of course, this speculation needed to be validated by the population-based study. The findings reported elsewhere suggested that more males than females were affected by ARTIs, which were not observed in our study.

Notably, our study occurred over a span of 3 years, which included the IAV (H1N1) outbreak in 2009. The impact of the outbreak on the results should be considered. Data showed that the detection rate of IAV increased significantly and co-infection rate during outbreak months was much higher than average co-infection rate. Unfortunately, we did not type these influenza strains based on the original study design. It was most likely that these strains contributed to the relatively high proportion of IAV. Relatively higher single and multiple infections of RSV, HRV and PIVs were also observed during the outbreak of IAV. Increased susceptible population and awareness, intensive testing and altered patient and physician behavior could lead to these increases. These factors could partly explain the relatively high proportion of pneumonia cases in the study. Furthermore, studies showed that the outbreak of IAV (H1N1) could increase the risk of other viral infections such as RSV and HRV.^24^,^33^ Other limitations also existed in this study. First, molecular methods allowed the detection of only viral nucleic acid even without virus replication, which complicates the interpretation of positive detection results. Second, the subtype identification of some common respiratory viruses such as IAV and HRV was not performed in our study, particularly during the IAV (H1N1) outbreak in 2009.

In summary, despite those aforementioned limitations, this three consecutive years' surveillance would provide a basic profile of the spectrum, seasonality, age and gender distribution, co-infection patterns as well as clinical association of viral respiratory infections in hospitalized children in the study sites. It could help the prediction, prevention and control of ARTIs in children.
